# Laxative effect of Zengye granule by modulating the SCF/c-Kit pathway and gut microbiota in constipated mice

**DOI:** 10.3389/fvets.2025.1628570

**Published:** 2025-06-18

**Authors:** Fengxia Lv, Pan Li, Bin Wang, Menglu Zhao, Peng Ji, Shishan Dong

**Affiliations:** ^1^College of Veterinary Medicine, Hebei Agricultural University, Baoding, China; ^2^Henan Muxiang Biological Co., Ltd., Zhengzhou, China; ^3^China–US (Henan) Hormel Cancer Institute, Zhengzhou, China; ^4^College of Veterinary Medicine, Gansu Agricultural University, Lanzhou, China

**Keywords:** Zengye granule, laxative effects, diphenoxylate, constipation, stem cell factor/c-kit, gut microbiota

## Abstract

**Introduction:**

Zengye granule (ZYG), a traditional Chinese medicine, is listed in the Chinese Pharmacopoeia as a prescription medicine for treating various yin-deficiency diseases including inner heat, dry mouth and pharynx, and dry bound stool. However, the underlying mechanisms of its action remain unclear. This study aimed to assess the laxative effects of ZYG on diphenoxylate-induced constipation in Kunming mice and clarify the underlying mechanism of action of ZYG in treating constipation.

**Methods:**

A model of constipation induced by diphenoxylate was developed. The laxative effect was evaluated based on the discharge time of the first black stool, fecal number, fecal weight, intestinal propulsion rate, and intestinal moisture content. Enzyme-linked immunosorbent assay was used to analyze the expression of inflammatory cytokines and neurotransmitters in serum. Histopathological analysis of colon tissues was performed using hematoxylin–eosin staining. Real-time quantitative polymerase chain reaction, immunohistochemistry, and western blotting were used to analyze the mRNA and protein expression of the stem cell factor (SCF)/c-Kit tyrosine kinase (c-Kit) signaling pathway. The composition of the mouse intestinal microbiota was determined by 16S rDNA sequencing.

**Results:**

ZYG improved intestinal peristalsis, defecation frequency, and intestinal moisture content. ZYG decreased the abundance of *Firmicutes* at the phylum and genus levels and increased the abundance of *Bacteroidetes* at the genus level. ZYG exerted a laxative effect by modulating the SCF/c-Kit signaling pathway.

**Discussion:**

This study provides valuable insights into laxative mechanism of ZYG and its potential veterinary application.

## 1 Introduction

Constipation impact patients’ quality of life because it is manifested as the reduced number of defecations, laborious defecation, and dry stool knot, requiring frequent usage of osmotic and stimulant laxatives (1). It is a highly common gastrointestinal disease in sows and females during late pregnancy and lactating period (2, 3). Although osmotic and stimulant laxatives can effectively control constipation symptoms in the short term, their long-term effects are poor (4) and can increase the risk of death (5), colon cancer (6, 7), and breast cancer (8). Therefore, the development of safe drugs and therapeutic regimens for constipation is crucial.

Traditional Chinese medicines (TCMs) are used in treating diseases, and several potent biologically active components of TCM are produced after metabolism by the intestinal flora. Some TCM formulae exert therapeutic effects against constipation through multiple components, targets, and mechanisms. For instance, the Ji-Chuan decoction can reduce enteric glial cell apoptosis in mice with slow transit constipation induced by diphenoxylate administration (9); Zengye decoction (ZYD) can regulate the intestinal microbiota and change the endogenous metabolite profiles in aged constipated rats (10); Li Qi Run Chang Fang can significantly increase the relative abundance of *Lactobacillus* and enhance the symbiotic relationships between *Lactobacillus* and other intestinal flora (11); MaZiRenWan, which includes dianthrones, anthraquinone glycosides, free anthraquinones, and other polyphenols, promotes gastrointestinal motility and relieve functional constipation (12); Da-Huang-GanCao-Tang shows an effective laxative effect owing to the presence of rhein 8-*O*-*β*-D-glucopyranoside that can change the function of *Bacteroides* and metabolize the prodrug Sennoside A (13, 14); Yangyin Tongmi capsule shows mitigative effects on diphenoxylate-induced constipation by regulating the content of intestinal hormones and neurotransmitters, and expression of related proteins in the colon (15); Zhizhu decoction shows laxative effect by activating the aryl hydrocarbon receptor signaling pathway and affecting the composition of gut microbiota in transit constipation mice (16). Therefore, some TCM formulae may offer considerable therapeutic effects against constipation.

ZYD, originating from volume II of “Wenbing Tiaobian” written by Wu Jutong during the Qing dynasty (AD 1636–1912), consists of four TCMs, such as Radix Scrophulariae (*Scrophularia ningpoensis* Hemsl., Chinese name Xuanshen; XS), Radix Ophiopogonis [*Ophiopogon japonicus* (Linn. f.) Ker-Gawl., Chinese name Maidong; MD], and Radix Rehmanniae (*Rehmannia glutinosa* Libosch., Chinese name Dihuang; DH), at a ratio of 1.0:0.8:0.8 (17, 18). ZYD has been commonly used for hundreds of years to tackle moisturizing dryness, promote the production of body fluids, and treat functional constipation associated with yin deficiency (17, 18). Zengye granule (ZYG), a prescription medicine based on ZYD, is listed in the Chinese Pharmacopoeia for treating various yin-deficiency diseases including inner heat, dry mouth and pharynx, and dry bound stool (19). We have previously reported that ZYG at a dose < 10 g·kg^−1^·day^−1^ for 1 day or 2.5 g·kg^−1^·day^−1^ for 30 consecutive days had no distinct toxicity or side effects in rats (20).

In this study, we aimed to investigate the laxative effects of ZYG in a mouse model of diphenoxylate-induced constipation and the underlying mechanism.

## 2 Materials and methods

### 2.1 Materials

Compound diphenoxylate (CD, diphenoxylate 2.5 mg and 0.025 mg atropine per tablet, batch: 1803008) was purchased from Changzhou Kangpu Pharmaceutical Co., Ltd. Ma Ren pills (MRP, batch: 20200403) were obtained from Wuhan Taifu Pharmaceutical Co., Ltd. Activated carbon powder (ACP) was purchased from Fuchen Chemistry and Reagent Co., Ltd. (Tianjin, China). Sterile saline (batch: C2230518A1) was supplied by Henan Kelun Pharmaceutical Co., Ltd.

### 2.2 ZYG preparation

ZYG (batch: 20200301) was produced by Henan Muxiang Veterinary Pharmaceutical Co., Ltd., following standard operating procedures, and strict quality control was maintained using high-performance liquid chromatography (HPLC), as previously described (20). As described by our pervious report, the harpagoside concentration in the ZYG (batch No.: 20200301) was 105.86 μg/g by using HPLC, indicating that the ZYG was good quality (20).

### 2.3 Materials and ZYG preparation for oral administration

The above materials and ZYG were freshly dissolved or suspended in sterile saline before gavage. ZYG was prepared at 0.6, 0.3, and 0.15 g/mL concentrations; CD was prepared at final concentrations of 2.5 mg/mL diphenoxylate and 0.025 mg atropine suspension; ACP was prepared as a 0.1 g/mL suspension; and MRP was prepared as a 0.12 g/mL suspension. Additionally, four mixed suspensions including ZYG (three final concentrations) and ACP, and MRP (one final concentration) and ACP were prepared at final concentrations of 0.1 g/mL ACP; 0.6, 0.3, and 0.15 g/mL ZYG; and 0.12 g/mL MRP.

### 2.4 Animals and grouping

A total of 120 male and 120 female specific pathogen free Kunming (KM) mice (20 g ± 2 g, 4–5–week-old) were purchased from Lanzhou Veterinary Research Institute Experimental Animal Center [SCXK (Gan)2020-0002]. All mice were housed under control conditions (temperature of 20–25°C, relative humidity of 40–70%, and a 12-h light/dark cycle) with regular chow and water freely available for 1-week adaptation. The animals were cared for in accordance with the *Laboratory Animal-Guideline for Ethical Review of Animal Welfare*. The study protocol was approved by the Ethics Committee of Gansu Agricultural University and was performed in accordance with the ethical standards (ethics approval number: GSAU-Eth-VMC-2024-222).

After fasting for 12 h with free access to water, body fluid deficiency constipation model of KM mice was generated by gavage of CD at doses of 50 mg/kg diphenoxylate and 0.5 mg/kg atropine (21, 22). After 30 min, mice were randomly divided into four experimental groups (*n* = 60) for measuring fecal parameters, activated carbon propulsion, and intestinal moisture contents, and hematologic, serum biochemical, and neurotransmitter analyses. Sixty KM mice were randomly divided into six groups (*n* = 10, five of each sex): control (group C), model (group M), MRP (group DC), low-dose ZYG (group ZL), medium-dose ZYG (group ZM), and high-dose ZYG (group ZH). Mice in groups C, M, and DC were orally administered (20 mL/kg) sterile saline, CD (50 mg/kg diphenoxylate and 0.5 mg/kg atropine), and 2.4 g/kg MRP, respectively, throughout the course of the study. Mice in groups ZL, ZM, and ZH were orally administered 3.0, 6.0, 12.0 g/kg (20 mL/kg) ZYG by gastric intubation, respectively. To study fecal parameters and activated carbon propulsion, mice in groups DC, ZL, ZM, and ZH were orally administered 2.0 g/kg ACP. All animals had *ad libitum* access to diet and drink.

### 2.5 *In vivo* laxative activity test

#### 2.5.1 Measurement of fecal parameters

After 30 min of ZYG administration, mice in each group were individually placed in metabolic cages for 8 h. The discharge time of the first black stool, fecal number, and fecal weight within 8 h were recorded.

#### 2.5.2 Activated carbon propulsion

Mice were euthanized 40 min after ZYG administration, and the small intestine was quickly removed. The total length of the small intestine (the distance from the pylorus to the ileocecal area) and distance traveled by activated carbon were measured. The activated carbon propulsion rate was calculated as Equation (1) (23):


(1) $$\text{Propulsion rate}=\frac{\text{Propulsive distance}}{\text{Total small intestine length}}\times 100\%$$

#### 2.5.3 Intestinal moisture content

Mice were euthanized 2 h after ZYG administration, and the small and large intestines were quickly removed. The weights of the small and large intestines were immediately recorded. Then, the intestines were dried at 90°C for 2 h. The percentage of intestinal moisture content was calculated as Equation (2) (23):


(2) $$\begin{array}{l} \text{Intestinal moisture content}\\ =\frac{\mathrm{Wet}\;\text{intestinal weight}-\mathrm{Dry}\;\text{intestinal weight}}{\mathrm{Wet}\;\text{intestinal weight}}\times 100\% \end{array}$$

#### 2.5.4 Hematological and serum biochemical analysis

After administration, mice in each group were maintained on the same diet and allowed to drink freely. General health and mortality were observed for 6 h. Immediately after the collection of red blood cells (RBC), hematological analysis was performed to evaluate RBC, white blood cell (WBC), and platelet (PLT) counts, and hemoglobin (Hb) content. For serum biochemical assay, blood samples in dry tubes were centrifuged at 3000 rpm for 15 min to separate serum. The Na^+^, K^+^, Cl^−^, and Ca^2+^ ion concentrations were measured using a BT-2000PLUS analyzer (Biotecnica Chemistry Co., Italy).

#### 2.5.5 Cytokine and neurotransmitter analysis

The levels of the neurotransmitters acetylcholine (Ach), nitric oxide synthase (NOS), substance P (SP), and vasoactive intestinal peptide (VIP), and those of proinflammatory cytokines interleukin (IL)-6, IL-1β, and tumor necrosis factor-alpha (TNF-*α*) in serum were determined using enzyme-linked immunosorbent assay kits, following the manufacturer’s instructions (Shanghai Enzyme-linked Biotechnology Co., Ltd., Shanghai, China).

#### 2.5.6 Hematoxylin–eosin (HE) staining

Colonic tissues were rinsed with normal saline, fixed in 4% paraformaldehyde, and embedded in paraffin. Paraffin-embedded tissues were dewaxed in water. They were subsequently sectioned and stained with HE (Beijing Solarbio Science & Technology Co., Ltd., Beijing, China). The sections were observed using an RX50 light microscope [Sunny Optical Technology (Group) Co., Ltd. Ningbo, China].

#### 2.5.7 Immunohistochemical (IHC) staining

Stem cell factor (SCF) and c-Kit tyrosine kinase (c-Kit) expression was measured in fixed colon tissues by IHC staining as previously described (24). Sections were incubated with DAB (ZLI-9018 2132A0325; Beijing Zhong Shan-Golden Bridge Biological Technology Co., Ltd., Beijing, China), and images were captured using an RX50 light microscope (Sunny Optical Technology (Group) Co., Ltd.). Image Pro Plus v.6.0 was used to measure fluorescence intensity.

#### 2.5.8 Real-time quantitative polymerase chain reaction (RTq–PCR) analysis

RTq–PCR was performed to quantify the mRNA expression of *SCF* and *c-Kit*. Total RNA from colonic tissues was extracted using TRIzol reagent, following the manufacturer’s instructions, as previously described (25). The results are presented as relative expression with respect to the internal control *β-actin*.

#### 2.5.9 Western blot analysis

Tissue samples were lysed using a radioimmunoprecipitation assay lysis buffer (Beijing Bioss Biotechnology Co., Ltd. Beijing, China) containing protease and phosphatase inhibitors, to extract proteins. Protein concentration was measured using a bicinchoninic acid protein assay kit (PC0020; Beijing Solarbio Science & Technology Co., Ltd.). Proteins were separated by 10% sodium dodecyl sulfate–polyacrylamide gel electrophoresis and transferred to a polyvinylidene difluoride membrane. The membrane was blocked with 8% skimmed milk and then incubated with primary antibodies at 4°C overnight. The membrane was then incubated with secondary antibodies for 2 h at room temperature. Finally, the membrane using AMERSHAM Image Quant 800 (Cytiva Co., Ltd. Shanghai, China). The primary antibodies (Beijing Biosynthesis Biotechnology Co., Ltd. Beijing, China), and the dilutions are as follows: c-Kit (1:100), SCF (1:200), and *β*-actin (1:1000). Protein expression is presented as relative value with respect to the internal control β-actin.

#### 2.5.10 Microbial DNA extraction and 16S rDNA gene sequencing

Genomic DNA from the microbial community was extracted using randomly selected fecal samples from each group. Gene sequencing and data analyses were performed as previously described (26).

### 2.6 Statistical analysis

The data are expressed as mean ± standard error. The data were compared using one-way analysis of variance followed by a *t*-test, using SPSS v.26.0 for Windows, to evaluate significant differences between groups. A *p*-value < 0.05 was considered significant.

## 3 Results

### 3.1 ZYG treatment improved symptoms of constipated mice

In this study, a constipation model was successfully induced in mice using diphenoxylate (group M). After oral administration of MRP and ZYG, the fundamental defecation indices (defecation frequency, oral-anal transit time, and fecal water content) of constipated mice significantly improved, and the constipation symptoms were alleviated.

#### 3.1.1 Fecal parameters

The fecal parameters are shown in Table 1. All mice in group C normally defecated. However, 60% (6/10), 20% (2/10), 20% (2/10), 10% (1/10), and 20% (2/10) mice did not defecate in groups M, DC, ZL, ZM, and ZH, respectively. Compared to that of the group M, the discharge time of the first black stool significantly decreased (*p* < 0.05), and the number, weight, and moisture content of feces in groups DC, ZL, ZM, and ZH significantly increased (*p* < 0.05). Compared to that of the group DC, the discharge time of the first black stool significantly decreased (*p* < 0.05) and the feces number significantly increased (*p* < 0.05) in groups ZM and ZH. The feces weight of group ZM was significantly higher than that of group DC. Therefore, ZYG has a superior laxative effect to MRP in constipated mice.

**Table 1:** Fecal parameters and activated carbon propulsion of KM mice treated with ZYG.

Fecal parameter	Group C	Group M	Group DC	Group ZL	Group ZM	Group ZH
No defecation mice	0	6	2	2	1	2
Discharge time of the first black stool (min)	139.70 ± 12.75^c^	303.50 ± 17.99^a^	191.75 ± 23.47^b^	170.75 ± 55.86^bc^	155.11 ± 50.25^c^	163.75 ± 14.49^bc^
Feces number	28.70 ± 5.46^a^	8.25 ± 2.36^d^	17.38 ± 2.39^c^	19.63 ± 5.37^bc^	22.78 ± 3.56^b^	20.63 ± 4.14^bc^
Feces weight (mg)	236.2 ± 29.14^a^	51.58 ± 17.07^c^	173.9 ± 15.1^b^	180.2 ± 33.7^b^	225.0 ± 21.5^a^	186.0 ± 18.2^b^
Propulsion (%)	70.63 ± 2.84^a^	50.06 ± 4.26^d^	60.12 ± 3.12%^c^	61.00 ± 3.28^bc^	63.68 ± 3.58^b^	60.85 ± 3.35^bc^

Values are mean ± standard error (*n* = 10). The different and same superscripts in the same list indicate statistical significance (*p* < 0.05) and no statistical significance (*p* > 0.05), respectively.

The time of the first black stool was shorter, and the number, weight, and moisture content of feces were higher in group ZM than those in group ZL and ZH. Notably, the time of the first black stool and weight of feces were not significantly different in groups ZM and C, indicating that mice in group ZM showed normal fecal parameters. This confirmed that ZYG administration at 6.0 g/kg had the best laxative effect.

#### 3.1.2 Activated carbon propulsion

Activated carbon propulsion is linked to the movement of the small intestine. Activated carbon propulsion of groups DC, ZL, ZM, and ZH was significantly higher (*p* < 0.05) than that of group M, and that of group ZM was significantly higher (*p* < 0.05) than that of group DC (Table 1). However, no significant differences in propulsion percentages were noticed between groups ZL and ZH. The level of activated carbon propulsion in the small intestine of mice group ZM was 63.68 ± 3.58%, which was higher than those of groups ZL and ZH. Activated carbon propulsion of group M was lowest (50.06 ± 4.26%). These results indicated that 6.0 g/kg ZYG significantly promoted peristalsis of the small intestine.

#### 3.1.3 Intestinal moisture contents

ZYG and MRP significantly increased (*p* < 0.05) the small and large intestinal moisture contents (Table 2). Notably, the large intestinal moisture contents significantly increased (*p* < 0.05) in group ZM compared to that in group C and DC. This indicated that 6.0 g/kg ZYG showed a superior laxative effect to MRP, and the intestinal moisture contents returned to normal levels.

**Table 2:** Intestinal moisture contents, and hematologic and serum biochemical analysis of KM mice treated with ZYG and MRP.

Parameter	Group C	Group M	Group DC	Group ZL	Group ZM	Group ZH
Intestinal moisture contents						
Large intestinal (%)	79.31 ± 1.32^bc^	78.20 ± 2.09^c^	79.63 ± 1.57^b^	80.71 ± 2.05^ab^	81.09 ± 0.98^a^	80.54 ± 1.15^ab^
Small intestinal (%)	78.97 ± 1.43^ab^	77.65 ± 1.12^c^	79.44 ± 1.61^a^	78.90 ± 2.71^ab^	80.22 ± 1.21^a^	78.92 ± 0.89^ab^
Hematology						
RBCs (10^12^/L)	8.72 ± 0.73^b^	8.12 ± 0.49^c^	9.52 ± 0.76^a^	9.27 ± 0.43^ab^	8.74 ± 0.69^b^	9.14 ± 0.56^ab^
PLT (10^9^/L)	977.10 ± 254.22^b^	938.80 ± 382.19^b^	1139.00 ± 368.79^ab^	1273.60 ± 271.44^a^	1058.60 ± 433.91^ab^	1109.10 ± 176.80^ab^
Hb (g/L)	141.00 ± 11.70^bcd^	132.60 ± 8.90^d^	155.80 ± 12.51^a^	149.70 ± 7.10^ab^	140.10 ± 13.09^cd^	146.50 ± 7.85^abc^
WBC (10^12^/L)	3.88 ± 0.69^a^	1.88 ± 0.80^c^	3.23 ± 1.94^ab^	2.40 ± 0.95^bc^	1.85 ± 0.56^c^	1.85 ± 0.66^c^
Serum biochemistry						
K^+^ (mmol/L)	8.38 ± 0.96	7.68 ± 0.71	8.47 ± 0.37	8.07 ± 0.76	8.33 ± 1.28	8.08 ± 1.17
Na^+^ (mmol/L)	149.20 ± 2.36^c^	151.16 ± 1.57^b^	152.64 ± 2.35^ab^	153.26 ± 1.54^a^	152.85 ± 1.23^a^	153.64 ± 1.82^a^
Cl^−^ (mmol/L)	107.81 ± 5.34	109.00 ± 1.30	108.84 ± 5.73	106.84 ± 7.17	110.63 ± 1.72	106.48 ± 7.05
Ca^2+^ (mmol/L)	1.42 ± 0.07^a^	1.28 ± 0.04^c^	1.35 ± 0.08^abc^	1.34 ± 0.13^bc^	1.36 ± 0.05^ab^	1.31 ± 0.10^bc^
Neurotransmitter						
Ach (μg/mL)	576.74 ± 26.95^a^	529.20 ± 24.70^d^	553.26 ± 22.51^b^	560.58 ± 11.54^ab^	548.12 ± 17.03^bc^	530.36 ± 11.51^cd^
NOS (μmol/L)	27.93 ± 0.56^d^	30.98 ± 1.05^a^	29.72 ± 1.00^b^	28.54 ± 0.72^cd^	28.78 ± 0.75^c^	28.89 ± 0.85^c^
SP (ng/L)	155.62 ± 6.78^b^	127.92 ± 5.97^c^	162.38 ± 7.62^a^	156.22 ± 4.31^ab^	159.49 ± 5.03^a^	161.15 ± 3.22^a^
VIP (ng/L)	91.32 ± 5.66^a^	70.42 ± 3.91^c^	86.33 ± 2.65^b^	89.18 ± 4.32^ab^	93.85 ± 4.88^a^	90.77 ± 5.12^a^
Inflammatory cytokines						
IL-1β (pg/mL)	81.41 ± 6.54^b^	106.18 ± 5.89^a^	78.33 ± 7.21^c^	83.39 ± 4.65^b^	79.75 ± 3.29^bc^	75.46 ± 5.18^c^
IL-6 (pg/mL)	71.38 ± 4.63^bc^	85.62 ± 6.85^a^	72.17 ± 4.21^b^	69.89 ± 3.66^c^	73.52 ± 5.86^b^	73.66 ± 3.71^b^
TNF-α (pg/mL)	449.15 ± 9.21^b^	482.19 ± 8.64^a^	419.63 ± 8.89^c^	428.55 ± 6.73^c^	441.79 ± 5.28^b^	446.32 ± 7.34^b^

Values are mean ± standard error (*n* = 10). The different and same superscripts in the same list indicate statistical significance (*p* < 0.05) and no statistical significance (*p* > 0.05), respectively.

### 3.2 Effect of ZYG treatment on hematologic parameter

Mice in the DC group showed the following significant changes (*p* < 0.05): (1) an increase in WBC counts compared to those in groups M, ZM, and ZH; (2) an increase in RBC counts compared to those in groups M and ZM; and (3) an increase in Hb concentration compared to those in groups C, M, and ZM (Table 2).

Mice in the ZYG treatment groups showed the following significant changes (*p* < 0.05) in hematological parameters (Table 2): (1) an increase in Hb concentration in group ZL compared to that in group ZM; and (2) an increase in PLT counts in group ZL compared to that in groups C and M.

These results indicated that 3.0 g/kg ZYG significantly improved blood circulation and treated inflammatory disorders. Notably, 3.0 g/kg ZYG showed the best effect on hematological indicators.

### 3.3 ZYG treatment improved serum biochemical parameters

#### 3.3.1 Ion concentration

The Na^+^ concentration in M and all treatment groups significantly increased (*p* < 0.05) compared to that in group C (Table 2). The Na^+^ concentrations in groups ZL, ZM, and ZH, and Ca^2+^ concentration in group ZM significantly increased (*p* < 0.05), compared to those in group M. No significant differences in K^+^ and Cl^−^ concentrations were noticed. These results indicated that ZYG significantly regulated the balance of Na^+^ and Ca^2+^ electrolytes.

#### 3.3.2 Neurotransmitter analysis

The Ach and NOS concentrations in group M significantly decreased (*p* < 0.05) and increased (*p* < 0.05), respectively, compared to those in group C (Table 2). After treatment with MRP and ZYG, the NOS concentrations in groups DC, ZL, ZM, and ZH significantly decreased (*p* < 0.05), and the Ach concentrations in groups DC, ZL, and ZM significantly increased (*p* < 0.05), compared to those in the M group. The Ach and NOS concentrations in constipated mice significantly decreased (*p* < 0.05) and increased (*p* < 0.05), respectively. Administration of MRP or ZYG significantly increased (*p* < 0.05) Ach concentration while decreasing (*p* < 0.05) concentration of NOS, showing a laxative effect.

The SP and VIP concentrations were significantly lower (*p* < 0.05) in group M than those in the other groups (Table 2). After treatment with MRP and ZYG, SP and VIP concentrations significantly increased (*p* < 0.05) compared to those in the M group (*p* < 0.05).

#### 3.3.3 Inflammatory cytokine analysis

The concentrations of IL-1β, IL-6, and TNF-*α* of group M were significantly higher (*p* < 0.05) than those of group C (Table 2). After MRP or ZYG treatment, the IL-1β, IL-6, and TNF-α levels significantly decreased (*p* < 0.05). This indicated that MRP and ZYG improved the microenvironment of the body, decreased inflammatory reactions, and maintained normality.

### 3.4 ZYG treatment restored the intestinal mucosal barrier

**Figure 1:**
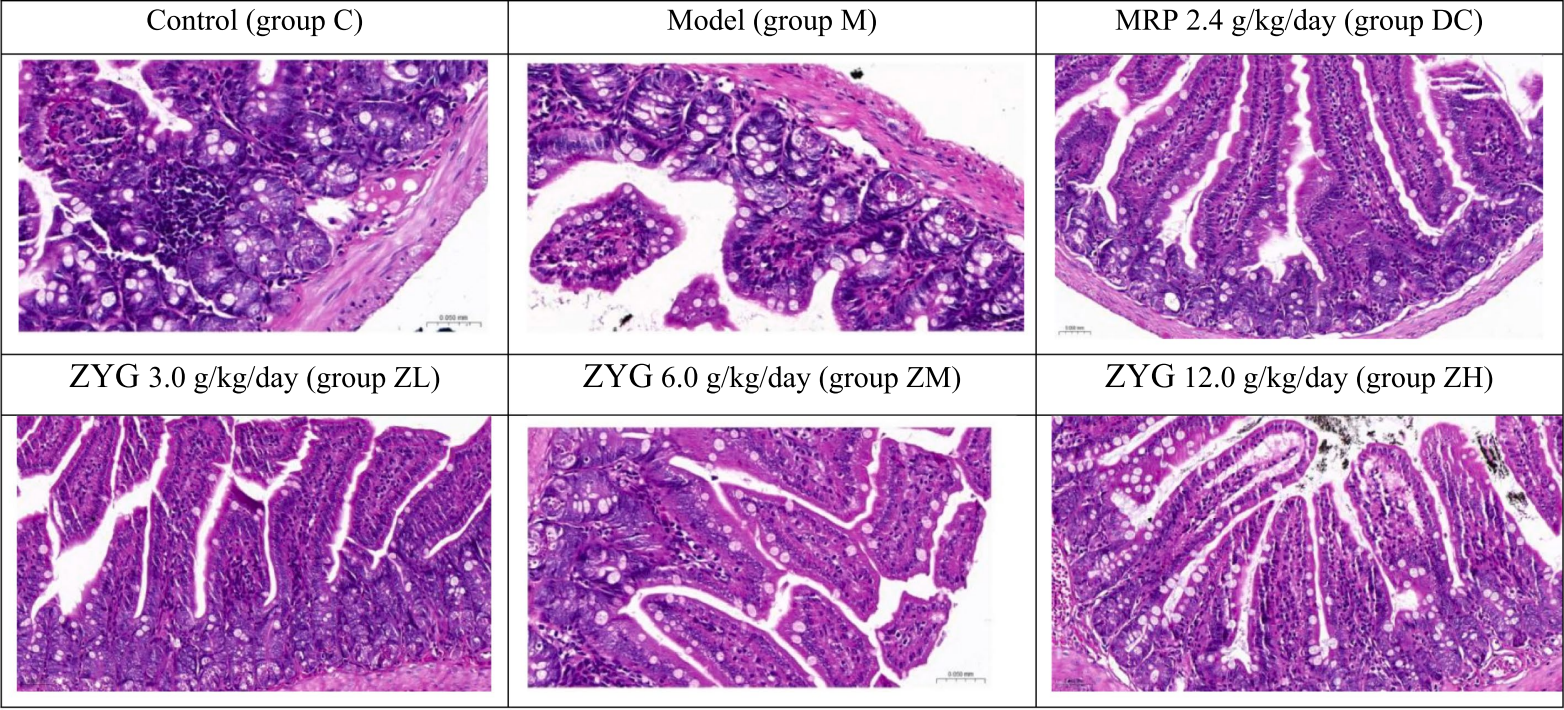
Histopathological analyses of colon tissues (HE, 400×).

Histopathological analyses of colon tissues (Figure 1) revealed the following significant findings in group M: (1) the epithelial mucosa on the tips of villi exfoliated, and mucosa were lost; (2) intercellular epithelium exhibited inflammatory cell infiltration; (3) Low number of goblet cells were observed; and (4) enteraden cells were lost. The colon organ tissues, including the mucosa, submucosa, smooth muscle layer, placenta, epithelium, and intestinal gland cells, in DC, ZL, ZM, and ZH groups showed no significant pathological differences in color or texture, compared with those in group C.

### 3.5 ZYG treatment upregulated the SCF/c-Kit signaling pathway

#### 3.5.1 Immunohistochemistry

**Figure 2:**
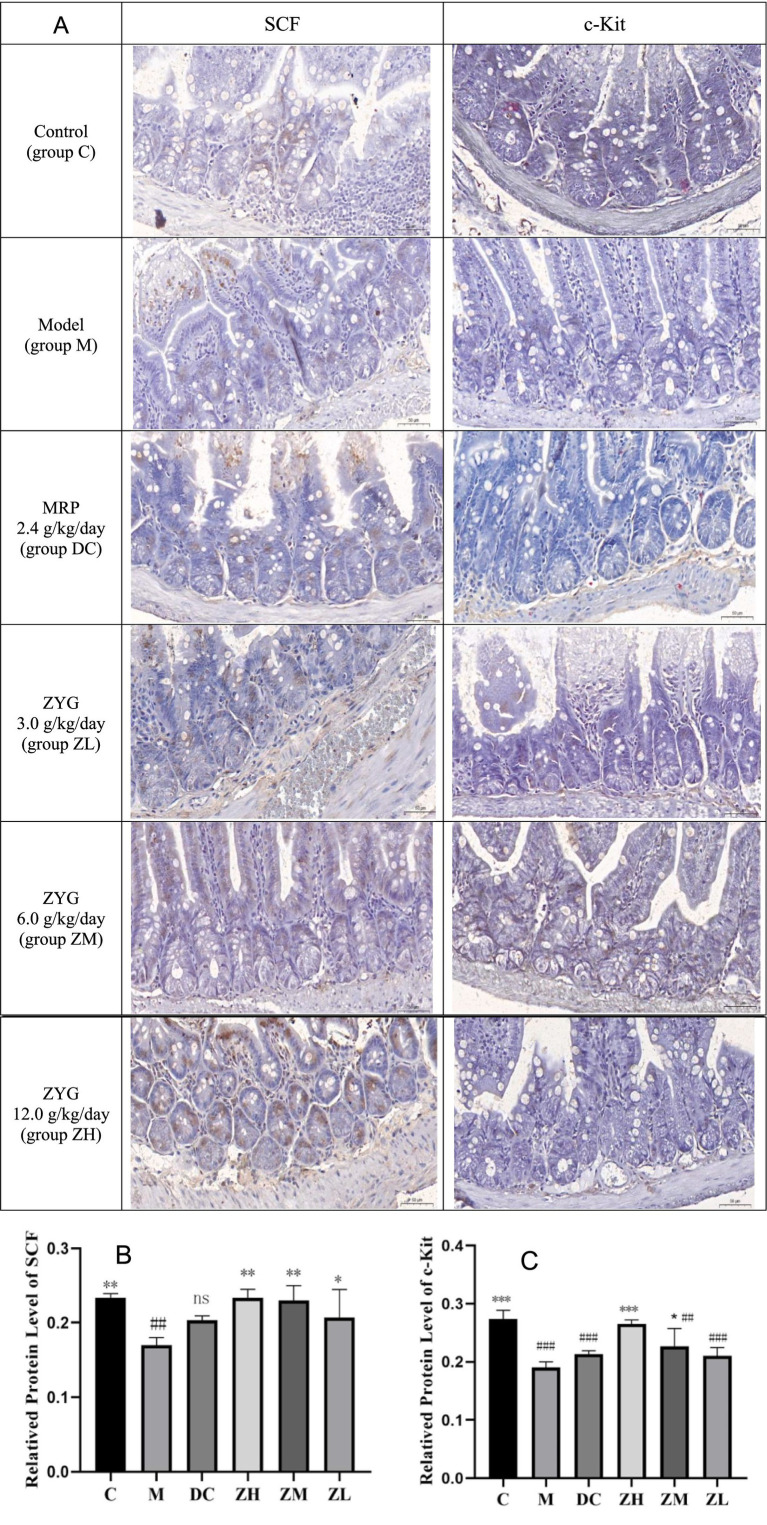
IHC analyses SCF and c-Kit (400×, **A**). Quantification of SCF **(B)** and c-Kit **(C)** levels in IHC analysis. Values are expressed as mean ± standard deviation (*n* = 10). The differences in SCF and c-Kit levels were analyzed using one-way analysis of variance (compared with the model group: ^*^p < 0.05, ^**^p < 0.01, ^***^p < 0.001; compared with the blank group: ^##^*p* < 0.01, ^###^*p* < 0.001).

SCF and c-Kit were stained as brown areas, as observed by light microscopy (Figure 2A). Staining intensity, area, and distribution were higher in group C than in groups M, DC, ZL, ZM, and ZH, and those in group M were lower than those in the other groups. SCF and c-Kit levels in group M were significantly downregulated compared to those in group C (Figures 2B,C). Their levels were significantly upregulated following MRP or ZYG intervention.

#### 3.5.2 mRNA expression

**Figure 3:**
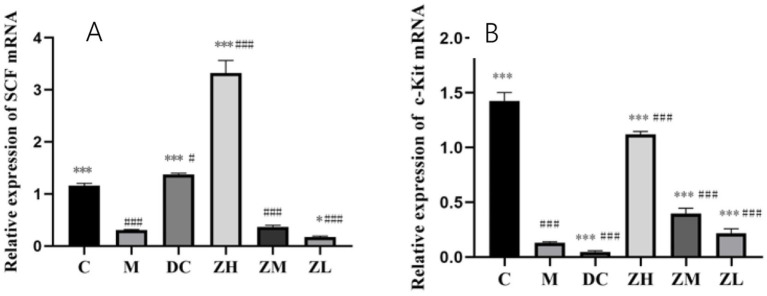
SCF **(A)** and c-Kit **(B)** mRNA expression after MRP and ZYG intervention. Values are expressed as mean ± standard deviation (*n* = 10). The differences in SCF and c-Kit levels were analyzed using one-way analysis of variance (compared with the model group: ^*^p < 0.05, ^***^p < 0.001; compared with the blank group: ^#^*p* < 0.05, ^###^*p* < 0.001).

The mRNA expression of *SCF* and *c-Kit* in group M was significantly downregulated (*p* < 0.001) compared to that in group C (Figures 3A,B). After treatment with MRP or ZYG, the mRNA expression of SCF showed the following significant changes: (1) it was significantly upregulated in groups ZH (*p* < 0.001) and DC (*p* < 0.05), compared to that in group C; (2) it was significantly upregulated (*p* < 0.001) in groups ZH, compared to that in group M; and (3) it was significantly upregulated (*p* < 0.001) in group DC compared to that in group M.

After treatment with MRP or ZYG, the mRNA expression of c-Kit showed the following significant changes: (1) it was significantly downregulated (*p* < 0.001) in group ZL compared to that in group C; and (2) it was significantly upregulated (*p* < 0.001) in groups ZL, ZM, and ZH compared to that in group M.

#### 3.5.3 Western blot analysis

**Figure 4:**
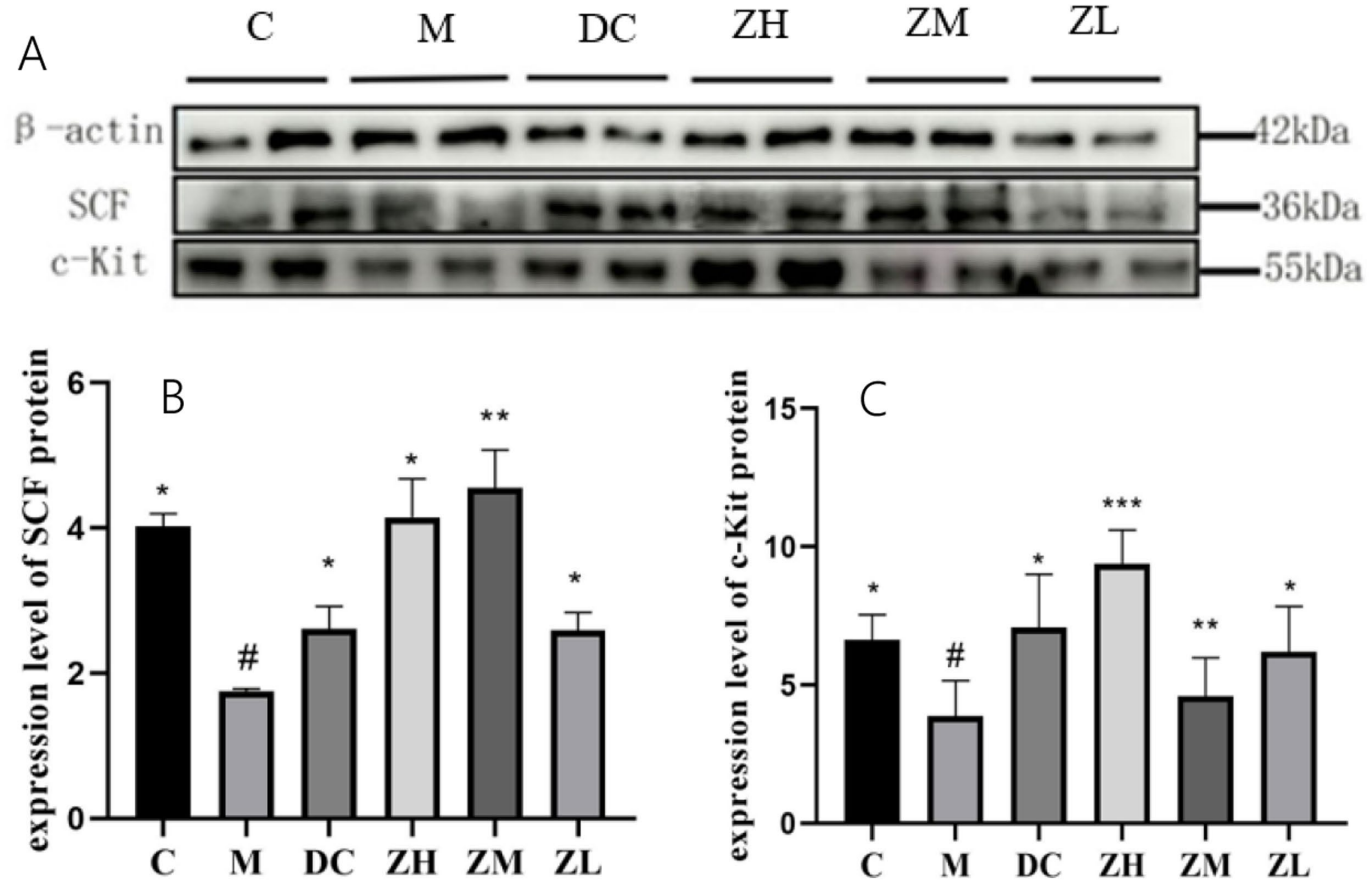
Western blot analysis of SCF and c-Kit expression **(A)** and quantitative analysis **(B,C)**. Values were expressed as mean ± standard deviation (*n* = 10). The differences in SCF and c-Kit levels were analyzed using one-way analysis of variance (compared with the model group: ^*^p < 0.05, ^**^p < 0.01, ^***^p < 0.001; compared with the blank group: ^#^*p* < 0.05, ^##^*p* < 0.01, ^###^*p* < 0.001).

SCF and c-Kit expression in group M significantly decreased (*p* < 0.05) compared to that in group C (Figures 4A–C). Mice treated with MRP or ZYG exhibited significant upregulation of SCF and c-Kit levels compared to that by mice in group M. These results indicate that the c-Kit/SCF signaling pathway was upregulated by ZYG treatment in constipated mice.

### 3.6 ZYG treatment modulated dysbiosis of gut microbiota

#### 3.6.1 Assessment of sequencing data quality and alpha diversity analysis

**Figure 5:**
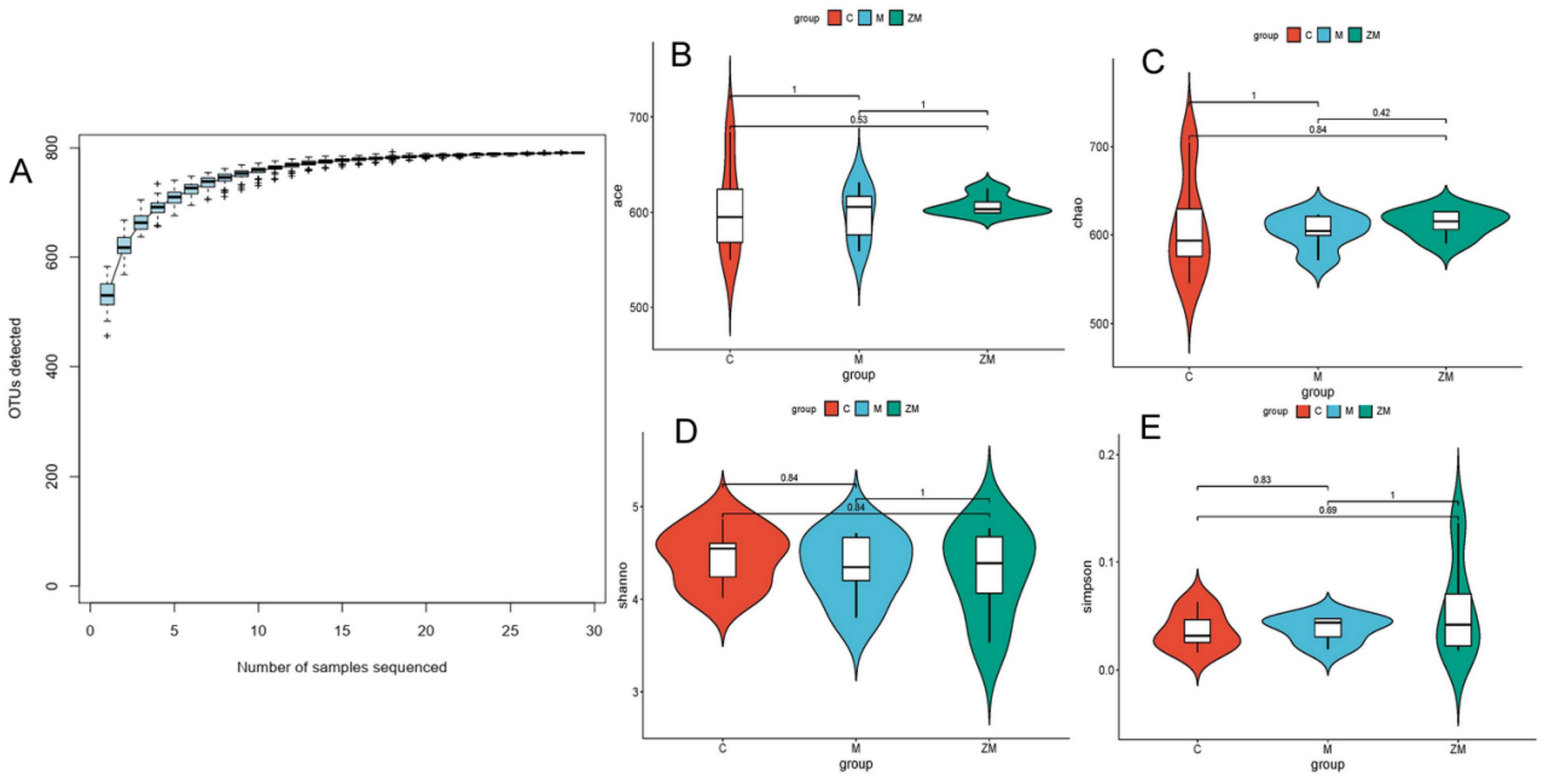
Accumulation curve of gut microbiota species **(A)**. Indices of ace **(B)**, Chao **(C)**, Shannon **(D)**, and Simpson **(E)**.

The sequencing amount was verified to reflect diversity of the original microorganisms. Alpha diversity analyses, including Chao, Ace, Shannon, and Simpson indices, were conducted. The rarefaction curve tended to be flat with an increase in the number of sampled sequences, indicating that the sequencing amount of each sample was sufficient (Figure 5A). Statistical analysis of the alpha diversity indices showed that the diversity of the different groups showed no obvious boundaries (*p* > 0.05; Figures 5B–E).

#### 3.6.2 Species annotation analysis

**Figure 6:**
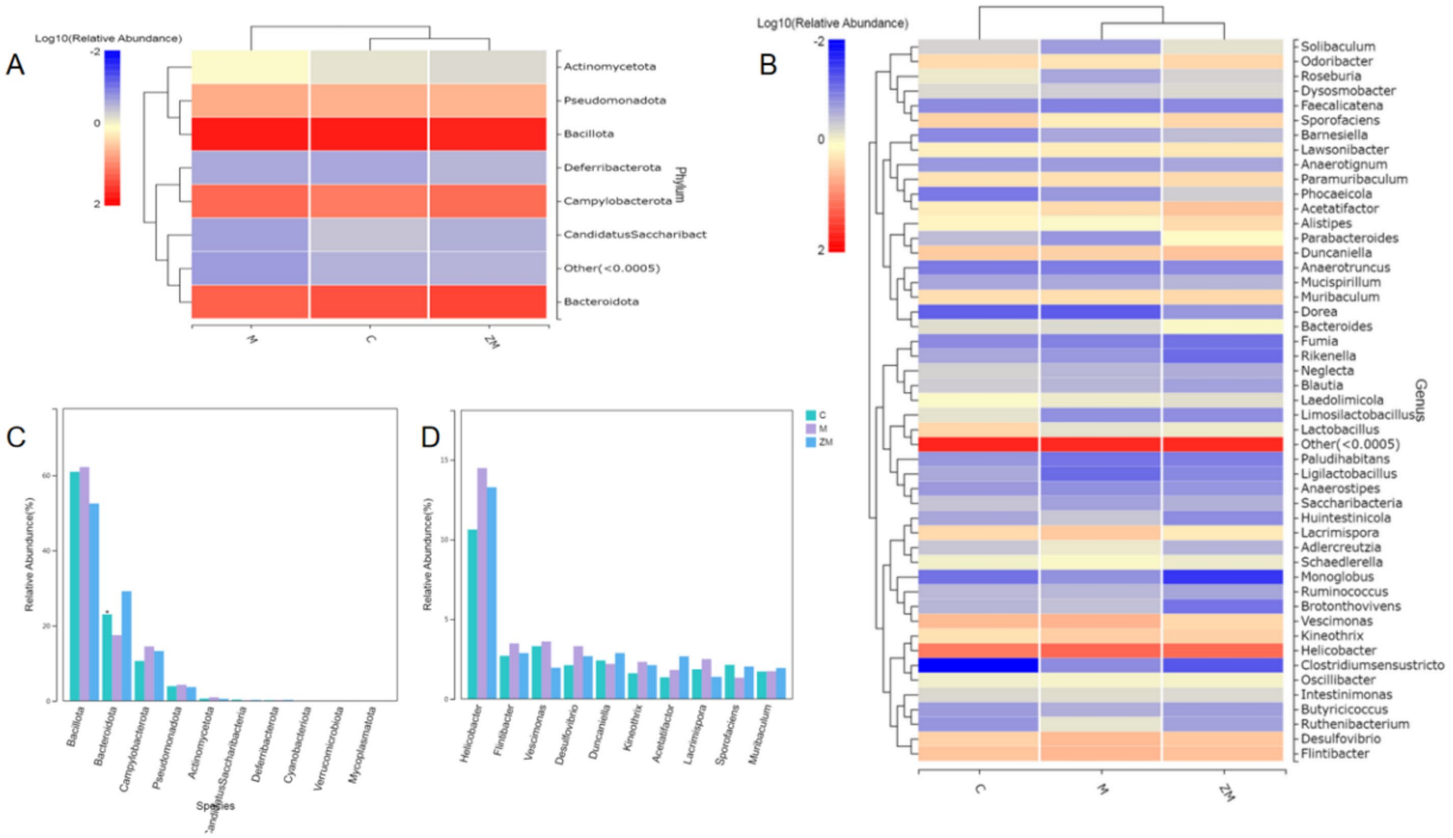
Taxonomic composition analysis of gut microbiota at the phylum and genus levels **(A)**. Phylum- and genus-level abundance distribution heatmap **(B)**. Comparison of microbial communities with differences at the phylum level **(C)**. Comparison of microbial communities with differences at the genus level **(D)**.

The results of species annotation analysis at the phylum and genus levels for groups C, M, and ZM are shown in Figures 6A,B. The predominant phyla in the gut microbiome of mice in these groups were *Firmicutes*, *Bacteroidota*, and *Campylobacterota*. At the phylum level, the abundances of *Firmicutes*, *Campylobacterota*, *Actinomycetota*, and *Pseudomonadota* were higher, whereas that of *Bacteroidetes* was lower in group M than in group C (Figure 6C). At the genus levels, the abundances of *Helicobacter*, *Flintibacter*, *Vescimonas*, *Desulfovibrio*, *Kineothrix*, *Acetatifactor*, and *Lacrimispora* were a higher, whereas those of *Duncaniella and Sporofaciens* were lower in group M than in group C (Figure 6D). After ZYG treatment, the phylum and genus levels were restored to those of group C. ZYG effectively restored the gut microbiota composition of constipated mice to the levels similar to those of group C.

#### 3.6.3 Analysis of significantly altered microbiota

**Figure 7:**
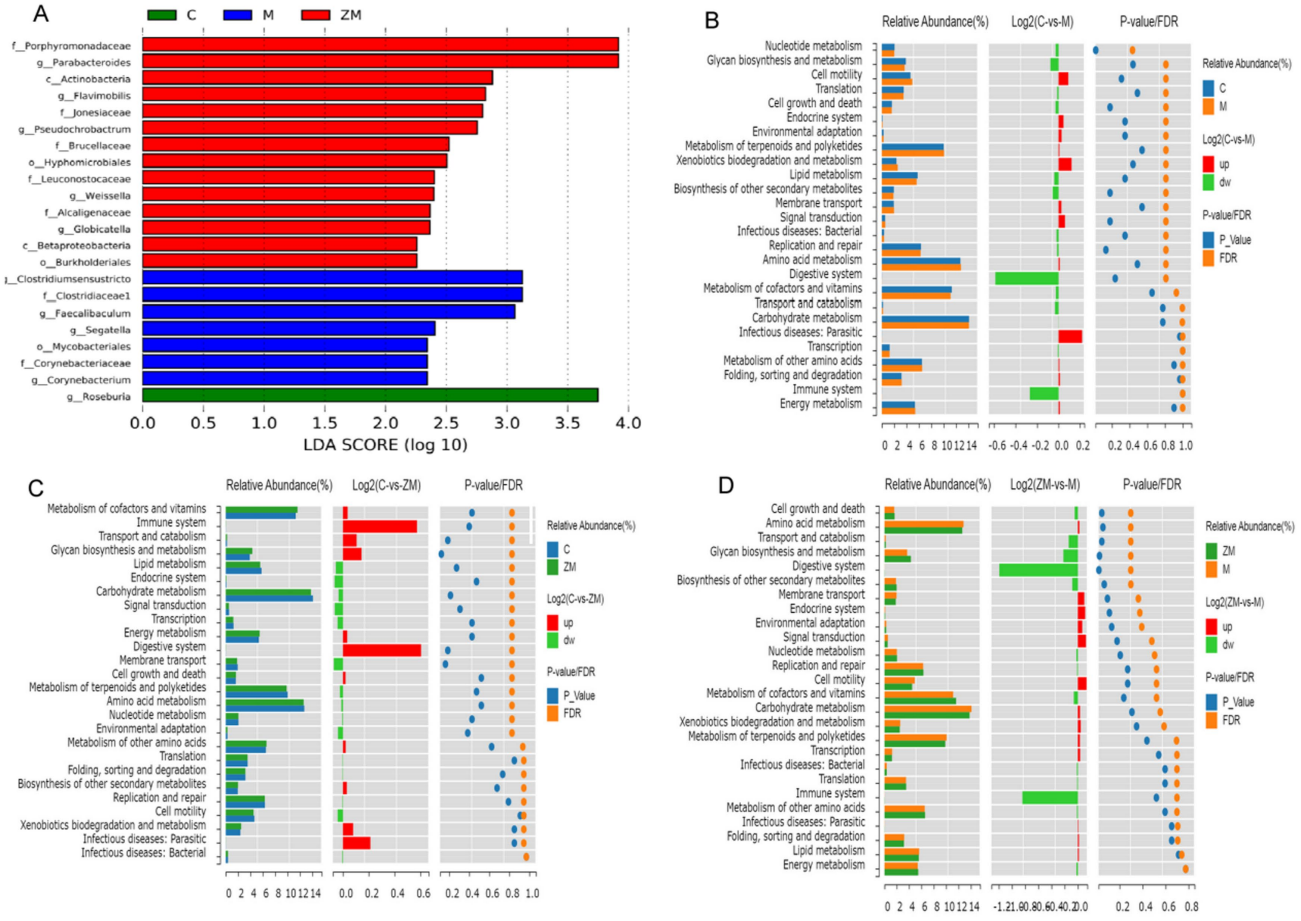
Linear discriminant analysis Effect Size analysis of gut microbiota: linear discriminant analysis histogram **(A)**. PICRUSt functional prediction analysis of DC and M **(B)**, DC and ZM **(C)**, and M and ZM **(D)** groups.

Linear discriminant analysis Effect Size was used to detect species that significantly differed among groups. The linear discriminant analysis scoring plot showed significant abundances of 22 microbes at the genus level in groups C, M, and ZM (Figure 7A). In group C, the significantly dominant gut microbiota was *Roseburia*. In group M, the significantly dominant gut microbiotas were *Clostridiumsensustricto*, *Mycobacteriales*, *Clostridiaceae*, *Segatella*, *Faecalibaculum*, *Corynebacteriaceae*, and *Corynebacterium*. In group ZM, the significantly dominant gut microbiotas were *Porphyromonadaceae*, *Parabacteroides*, *Actinobacteria*, *Flavimobilis*, *Jonesiacea*, *Pseudochrobactrum*, *Brucellaceae*, *Hyphomicrobiales*, *Leuconostocaceae*, *Weissella*, *Alcaligenaceae*, *Globicatella*, *Betaproteobacteria*, and *Burkholderiales.*

#### 3.6.4 Functional prediction of differential microbes

To study the specific functions of the gut microbiota, PICRUSt was used to predict the metabolic function profiles of ZYG at the genus level (Figures 7B–D). These differential microbes were highly related to nucleotide and amino acid metabolism, glycan biosynthesis and metabolism, energy metabolism, cell motility, growth, and death, immune and endocrine systems, carbohydrate and lipid metabolism, and digestive system.

## 4 Discussion

ZYD is a classic TCM formula comprising XS, SD, and MD, which can increase fluid and moistening dryness. XS is used as a “monarch drug” for nourishing Yin, and SD and MD are used as “ministerial drug” in this formula.

Constipation is characterized by infrequent bowel movements, and difficulty during or incompletion of defecation, which is caused by decreased peristaltic ability of the small intestine (4). Additionally, the reduction in peristalsis of the intestinal smooth muscle can increase water absorption, leading to reduced stool moisture and dry stools. The intestinal propulsive rate, quantity, and water content of feces are key indices of constipation, which are used to evaluate the laxative effect of drugs (27). The diphenoxylate-induced constipation model has been widely used to study the efficacy and mechanism of laxatives, which result in fluid deficiency and reduce the moisture content of the intestine and feces. MRP is a TCM formula comprising six Chinese herbs including Semen cannabis, Rhubarb, Apricot kernel, *Magnolia officinalis*, *Paeonia lactiflora* Pall, and Fructus aurantii immaturus (28). MRP is commonly used to treat chronic constipation by regulating the intestinal flora, improving intestinal movement, and alleviating constipation symptoms (29, 30). In the present study, MRP was used as a positive control for assessing the potential of ZYG as a therapeutic drug for treating constipation. ZYG restored normal fecal parameters in constipated mice and significantly promoted the peristaltic ability of the small intestine. Notably, 6.0 g/kg ZYG offered more effective constipation therapy than did 2.4 g/kg MRP. Consumption of dietary fiber (2), probiotics (3, 31), inulin and isomalto-oligosaccharide (32), and sugarcane bagasse (33) can relieve constipation in sows. Therefore, further studies are necessary to evaluate the therapeutic effects of ZYG in constipated sows.

Hematological parameters can serve as supporting evidence of renal function. In this study, the hematological parameters significantly increased by ZYG treatment, indicating that ZYG could restore normal renal function in constipated mice.

The enteric nervous system (ENS) is at the core of the regulatory control and defensive functions of the digestive tract, which is jointly constituted by excitatory and inhibitory nerves (34, 35). Neural signals pass between distinct gut regions for coordinating digestive activity using a wide range of chemical messengers. Ach, an excitatory neurotransmitter in the ENS, can directly activate specific receptors on the membrane of gastrointestinal smooth muscle and gland cells, and promote intestinal peristalsis (36, 37). SP, an excitatory neurotransmitter, can activate Ach and induce smooth muscle contractions, thereby acting as a regulator of Ach (38). In contrast, VIP, an inhibitory neurotransmitter, exhibits a strong vasodilatory effect, specifically by inhibiting the tone of intestinal muscles (39, 40). Excessive NOS production in the ENS typically inhibits intestinal contractions, thereby contributing to constipation (41). ZYG treatment effectively improved constipation symptoms by significantly regulating the expression of Ach, NOS, SP, and VIP in constipated mice.

Serum inflammatory cytokine levels positively correlate with the severity of constipation, which can increase intestinal permeability and lead to impaired intestinal mucosal barrier function (42). We noticed that ZYG treatment decreased the levels of IL-1β, IL-6, and TNF-*α*, reduced colonic inflammation, maintained the colonic structure, and alleviated histopathological deterioration in constipated mice.

The abundance of beneficial bacteria and levels of pathogenic bacteria or conditioned pathogens in the intestine are reduced and increased, respectively, by constipation, which can cause displacement of intestinal bacteria, leading to the release of several inflammatory factors (11). *Bacteroidetes* and *Firmicutes* are the most abundant phyla in the gut microbiota, and the ratio of *Firmicutes* to *Bacteroidetes* (F/B) is an index for evaluating intestinal health. An increased F/B ratio can lead to an increased risk of intestinal diseases (43–45). ZYD can reduce the levels of harmful bacteria and increase the abundance of beneficial bacteria (10). We noticed that ZYG significantly increased the phylum level of *Bacteroidetes*, indicating that the F/B ratio significantly increased and reduced the amounts of harmful metabolites in the body. ZYG effectively restored constipated mice to normalcy by regulating the expression of several neurotransmitters, inflammatory cytokines, and the gut microbiota.

The proliferation of interstitial cells of Cajal (ICC) is regulated by c-Kit and SCF, which are closely related to gastrointestinal motility (46, 47). Therefore, modulating ICC by targeting the SCF/c-Kit signaling pathway may be a potential therapy for constipation. Some TCMs and their formulae, such as *Cistanche deserticola* (48), *Prunus persica* (L.) Batsch blossom (25), and Qi Lang (49), can promote ICC proliferation by targeting the SCF/c-Kit signaling pathway for relieving constipation. Our study showed that ZYG promoted intestinal motility in diphenoxylate-induced constipated mice by significantly upregulating mRNA and protein expression of SCF and c-Kit.

In summary, ZYG shows excellent therapeutic effect in alleviating constipation in diphenoxylate-treated mice. It is necessary to evaluate the therapeutic effect of ZYG in constipated model sows. Therefore, this study provides a scientific basis for the veterinary clinical application of ZYG for treating constipation in sows.

## Data Availability

The original contributions presented in the study are included in the article/Supplementary material, further inquiries can be directed to the corresponding authors.

## Glossary

TCM: Traditional Chinese medicine
ZYD: Zengye decoction
XS: Radix Scrophulariae
MD: Radix Ophiopogonis
DH: Radix Rehmanniae
ZYG: Zengye granule
CD: Compound diphenoxylate
MRP: Ma Ren pills
ACP: Activated carbon powder
HPLC: High-performance liquid chromatography
KM: Kunming mice
RBC: Red blood cell
WBC: White blood cell
PLT: Platelet count
Hb: Hemoglobin
Ach: Acetylcholine
NOS: Nitric oxide synthase
SP: Substance P
VIP: Vasoactive intestinal peptide
IL: Cytokines interleukin
TNF-α: Tumor necrosis factor-alpha
HE: Hematoxylin–eosin
IHC: Immunohistochemistry
SCF: Stem Cell Factor
c-Kit: c-Kit tyrosine kinase
RTq–PCR: Quantitative real-time polymerase chain reaction
ENS: Enteric nervous system
ICC: Interstitial cells of Cajal
