# Supplementary material for: Laxative effect of Zengye granule by modulating the SCF/c-Kit pathway and gut microbiota in constipated mice
Source: Front Vet Sci. 2025 Jun 18;12:1628570. doi: 10.3389/fvets.2025.1628570 (PMC12217940; doi:10.3389/fvets.2025.1628570)
Supplement: Supplementary file 1 [file Presentation_1.pptx]

## Slide 1
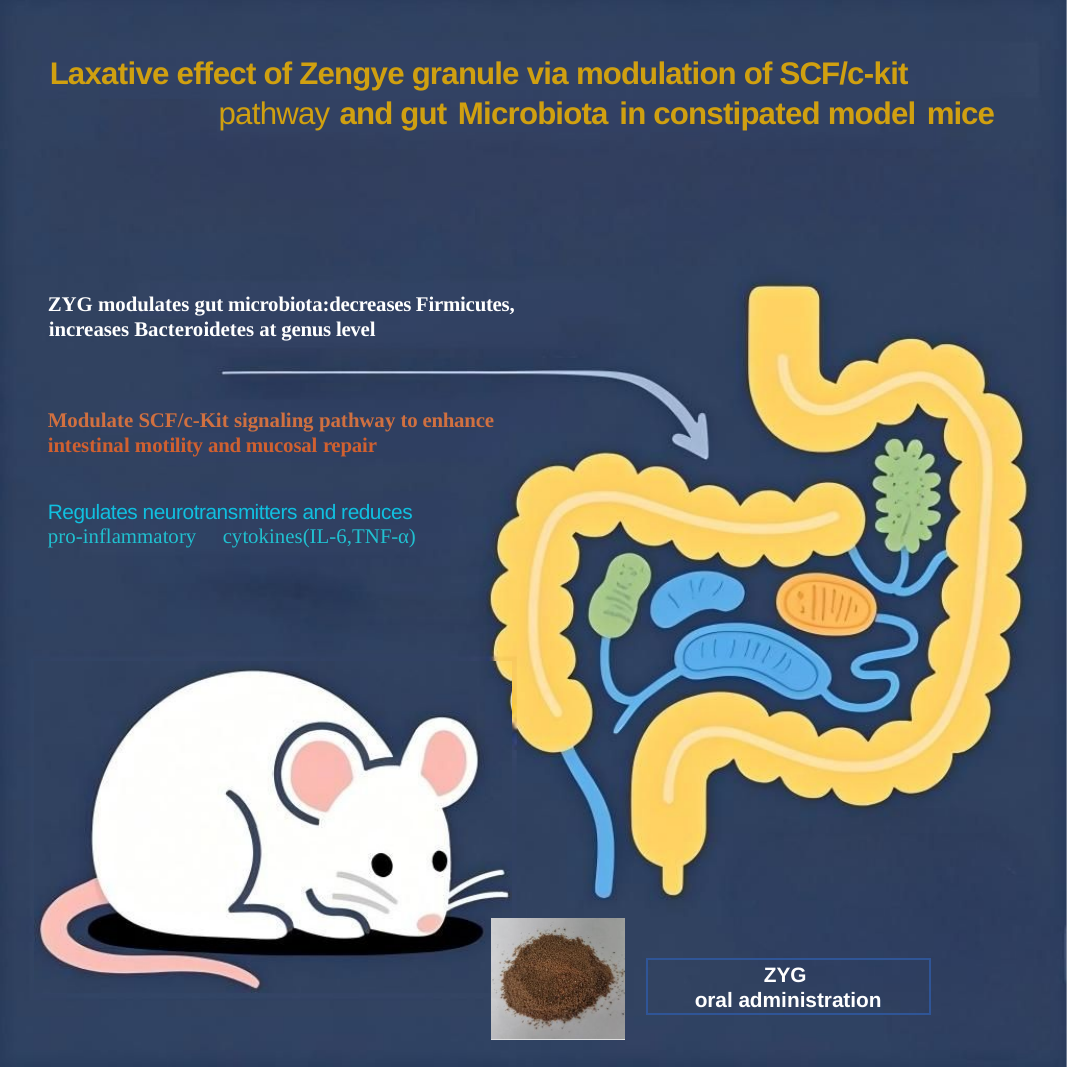

Laxative effect of Zengye granule via modulation of SCF/c-kit pathway and gut Microbiota in constipated model mice
ZYG modulates gut microbiota:decreases Firmicutes,
increases Bacteroidetes at genus level
Modulate SCF/c-Kit signaling pathway to enhance
intestinal motility and mucosal repair
Regulates neurotransmitters and reduces
pro-inflammatory cytokines(IL-6,TNF-α)
ZYG
oral administration
